# Colposcopic evaluation of unhealthy cervix in gynecology patients: A prospective observational study

**DOI:** 10.6026/973206300212001

**Published:** 2025-07-31

**Authors:** Archana Kori, Mohini Ahirwar, Jyoti Meravi, Manik Sirpurkar, Abhay Kumar

**Affiliations:** 1 Department of Obstetrics and Gynecology, Chhindwara Institute of Medical Sciences, Chhindwara, Madhya Pradesh, India; 2 Department of Obstetrics and Gynecology, Atal Bihari Vajpayee Government Medical College, Vidisha, Madhya Pradesh, India; 3 Department of ENT, Chhindwara Institute of Medical Sciences, Chhindwara, Madhya Pradesh, India

**Keywords:** Colposcopy, unhealthy cervix, cervical intraepithelial neoplasia, pap smear, cervical cancer screening, histopathology

## Abstract

Cervical cancer remains a major concern, especially in developing countries, highlighting the need for effective screening. In this study of 150 women with clinically unhealthy cervix, colposcopy showed higher sensitivity (83.3%) but lower specificity (72.7%) compared to Pap smear. Colposcopy-directed biopsy confirmed CIN in 21.3% of cases. The correlation between colposcopy and histopathology was 90.9%. Combining colposcopy with cytology enhances early detection of cervical lesions.

## Background:

Cervical cancer is the second most common cancer among women worldwide, with a disproportionately high burden in developing countries [1]. In India, it ranks as the second leading cancer in women aged 15-45 years, contributing to approximately 14% of all female cancer cases [2]. Despite being largely preventable through effective screening and early intervention, cervical cancer continues to cause significant morbidity and mortality due to inadequate screening coverage and poor follow-up, especially in low-resource settings [3]. The progression of cervical cancer follows a well-established pathway, beginning with a pre-invasive stage characterized by cervical intraepithelial neoplasia (CIN), which can evolve into invasive carcinoma over 10-15 years [1, 4]. This extended pre-invasive phase offers a critical window for early detection and treatment, making cervical cancer one of the most preventable malignancies [5]. Countries with organized screening programs have witnessed substantial reductions in cervical cancer incidence and mortality, highlighting the importance of early detection strategies [3]. While the Pap smear has been the cornerstone of cervical cancer screening, it has notable limitations, including variable sensitivity (30-87%) and high false-negative rates (15-50%) [6]. These challenges have prompted the integration of alternative diagnostic methods, with colposcopy emerging as a valuable adjunctive tool [7]. Colposcopy enables magnified visualization (7.5-15x) of the cervix, vagina and vulva, enhancing the detection of precancerous lesions [8]. The application of acetic acid and Lugol's iodine during the procedure highlights abnormal epithelial areas, allowing for targeted biopsies [9]. This approach improves diagnostic accuracy compared to reliance on cytology alone, particularly in women presenting with clinically "unhealthy cervix"-a term encompassing cervical erosion, cervicitis, hyperemia, contact bleeding, leukoplakia and suspicious growths observed during gynecological examination [5, 10]. Women with unhealthy cervix frequently report symptoms such as abnormal vaginal discharge, postcoital bleeding, or intermenstrual bleeding, which, although nonspecific, may indicate underlying premalignant or malignant pathology [5]. Historically, empirical treatment without detailed evaluation was common, risking missed diagnoses of significant lesions [6]. Recent evidence supports a structured diagnostic approach incorporating cytology, colposcopy and biopsy for accurate assessment and management [7, 9]. Colposcopy plays a critical role in identifying abnormal vascular patterns, acetowhite changes and atypical transformation zones suggestive of dysplasia [3, 8]. Although correlation rates between Colposcopic findings and histopathology vary (67%-95%), colposcopy remains a cornerstone in cervical pathology assessment when performed by experienced clinicians [4, 9]. In resource-limited settings, opportunistic screening using colposcopy for symptomatic women or those with unhealthy cervix offers a pragmatic solution to improve early detection rates [7, 10]. However, standardized protocols and comparative data on colposcopy versus cytology in this context are limited [9]. Therefore, it is of interest to evaluate the diagnostic performance of colposcopy in detecting cervical abnormalities among women with clinically unhealthy cervix, correlating findings with cytology and histopathology and determining its sensitivity, specificity and predictive values.

## Materials and Methods:

The study received approval from the institutional ethics committee [CIMS/EC/2022/6399] at a single center before its commencement. Participating subjects granted written approval to participate in the study.

## Study population:

Women aged 25-60 years attending the gynecology outpatient department at Chhindwara Institute of Medical Sciences, Chhindwara, MP, India, from January 2024 to January 2025, with symptoms such as abnormal vaginal discharge, postcoital bleeding and intermenstrual bleeding, or with clinically unhealthy cervix on speculum examination were considered for inclusion in the study. Clinically unhealthy cervix was defined as the presence of one or more of the following findings: cervical erosion, cervicitis, hyperemia, contact bleeding, leukoplakia, or suspicious growth.

## Exclusion criteria:

The following patients were excluded from the study: women with active vaginal bleeding, pregnant women, women with previously diagnosed and treated cervical cancer, women who had undergone hysterectomy and women who did not consent to participate in the study.

## Sample size calculation:

Based on previous studies reporting the prevalence of cervical abnormalities in women with unhealthy cervix to be approximately 30%, a sample size of 150 was calculated to estimate the true prevalence with a precision of 7.5% and a confidence level of 95%, accounting for a 10% non-response rate.

## Data collection and procedures:

A detailed history including demographic data, obstetric history and menstrual history and presenting symptoms was obtained from all participants using a structured questionnaire. All participants underwent a thorough gynecological examination, including speculum examination to assess cervical appearance. Pap smear was performed for all participants using conventional methods. Cervical samples were collected using an Ayre's spatula and endocervical brush and the material was spread on glass slides, fixed with 95% ethanol and sent for cytological examination. The results were interpreted according to the 2014 Bethesda System for reporting cervical cytology. Colposcopic examination was performed for all participants regardless of Pap smear results. The colposcopic procedure was carried out using a digital video colposcope (Model ASCON AC3-2000SN with green filter, focal length 250 mm and magnification 7.5X-10X) by an experienced gynecologist trained in colposcopy. The examination was performed according to standard protocol, including naked eye examination, examination after application of 5% acetic acid and application of Lugol's iodine (Schiller's test). Colposcopic findings were documented using standardized nomenclature according to the 2011 International Federation for Cervical Pathology and Colposcopy (IFCPC) classification. The colposcopic impression was categorized as normal, abnormal (minor changes, major changes, or suspicious for invasion), or unsatisfactory.

Colposcopy-directed biopsy was performed for all patients with abnormal colposcopic findings. Multiple biopsies (2-4) were taken from the most suspicious areas identified during colposcopy. The biopsy specimens were fixed in 10% formalin and sent for histopathological examination. The histopathological diagnosis was categorized as normal/inflammatory, CIN I, CIN II, CIN III, or invasive carcinoma.

## Statistical analysis:

Data were analyzed using SPSS version 25.0 (IBM Corp., Armonk, NY, USA). Descriptive statistics were presented as frequencies, percentages, means and standard deviations. The diagnostic performance of colposcopy was evaluated by calculating sensitivity, specificity, positive predictive value (PPV), negative predictive value (NPV) and accuracy, using histopathology as the gold standard. The correlation between colposcopic findings and histopathological diagnosis was assessed using the kappa statistic. A p-value of <0.05 was considered statistically significant.

## Results:

A total of 150 women with clinically unhealthy cervix were included in the study. The mean age of participants was 35.4±7.8 years (range: 25-59 years). The demographic and clinical characteristics of the study population are presented in Table 1 (see PDF). The majority of participants (40.0%) were in the 30-39 years age group and most had parity of 1-2 (50.7%). The most common presenting symptom was vaginal discharge (65.3%), followed by lower abdominal pain (30.0%). Cervical erosion was the most frequent clinical finding (58.0%), followed by cervicitis (28.0%). The results of Pap smear, colposcopy and histopathological examination are summarized in Table 2 (see PDF). ASCUS: Atypical squamous cells of undetermined significance; LSIL: Low-grade squamous intraepithelial lesion; HSIL: High-grade squamous intraepithelial lesion; CIN: Cervical intraepithelial neoplasia. Of the 150 participants, 56.0% had normal Pap smear results, while 25.3% showed inflammatory changes. Abnormal cytology was reported in 28 patients (18.7%), with ASCUS in 8.0%, LSIL in 6.7%, HSIL in 3.3% and squamous cell carcinoma in 0.7%. Colposcopic examination revealed normal findings in 71 patients (47.3%), abnormal findings in 64 patients (42.7%) and unsatisfactory colposcopy in 15 patients (10.0%). Among the patients with abnormal colposcopic findings, 39 (26.0%) had minor changes (Grade 1), 22 (14.7%) had major changes (Grade 2) and 3 (2.0%) had findings suspicious for invasion. All 64 patients with abnormal colposcopic findings underwent colposcopy-directed biopsy. Histopathological examination confirmed chronic cervicitis in 30 patients (46.9%), CIN I in 17 patients (26.6%), CIN II in 10 patients (15.6%), CIN III in 5 patients (7.8%) and invasive carcinoma in 2 patients (3.1%). The correlation between Pap smear, colposcopy and histopathology findings is presented in Table 3 (see PDF). ASCUS: Atypical squamous cells of undetermined significance; LSIL: Low-grade squamous intraepithelial lesion; HSIL: High-grade squamous intraepithelial lesion; SCC: Squamous cell carcinoma; CIN: Cervical intraepithelial neoplasia. Among the 25 patients with normal or inflammatory Pap smear who underwent biopsy due to abnormal colposcopic findings, 2 (8.0%) were found to have CIN I on histopathology. Of the 12 patients with ASCUS, 7 (58.3%) had CIN I or higher lesions on histopathology. All patients with HSIL or SCC on Pap smear had confirmed precancerous or cancerous lesions on histopathology. Regarding colposcopic findings, among the 39 patients with minor changes, 11 (28.2%) had CIN I or higher lesions on histopathology. Among the 22 patients with major changes, 20 (90.9%) had CIN I or higher lesions. All three patients with colposcopic findings suspicious for invasion had CIN III or invasive carcinoma on histopathology. The diagnostic performance of Pap smear and colposcopy in detecting cervical precancerous and cancerous lesions, using histopathology as the gold standard, is presented in Table 4 (see PDF). The sensitivity of colposcopy (83.3%) was significantly higher than that of Pap smear (54.3%) in detecting cervical precancerous and cancerous lesions. However, the specificity of colposcopy (72.7%) was lower than that of Pap smear (96.2%). The positive predictive value was slightly higher for Pap smear (91.7%) compared to colposcopy (88.9%), while the negative predictive value was higher for Pap smear (73.9%) compared to colposcopy (64.0%). The overall accuracy was higher for colposcopy (82.7%) compared to Pap smear (79.3%). The agreement between colposcopic impression and histopathological diagnosis was assessed using the kappa statistic, which showed a substantial agreement (κ = 0.78, p < 0.001).

## Discussion:

This prospective study evaluated the effectiveness of colposcopy in detecting cervical abnormalities in women with clinically unhealthy cervix, correlating colposcopic findings with cytology and histopathology. Our findings demonstrate that colposcopy has higher sensitivity but lower specificity compared to Pap smear in detecting cervical precancerous and cancerous lesions, supporting the complementary role of both screening methods in evaluating women with an unhealthy cervix. The majority of participants (40.0%) were in the 30-39 years age group, aligning with previous studies reporting a mean age of approximately 34 years for women with unhealthy cervix [5]. This reflects the peak incidence of cervical intraepithelial neoplasia (CIN), typically occurring in the third and fourth decades of life [6, 7]. Vaginal discharge was the most common presenting symptom (65.3%), followed by lower abdominal pain (30.0%); consistent with earlier reports identifying white discharge as the predominant complaint among women with unhealthy cervix [8]. These nonspecific symptoms often lead to opportunistic screening for cervical abnormalities [9]. Cervical erosion (58.0%) was the most frequent clinical finding, followed by cervicitis (28.0%), similar to previous observations where cervical erosion was predominant in women with unhealthy cervix [6, 10]. However, such clinical signs alone are insufficient to predict CIN, emphasizing the importance of cytology, colposcopy and biopsy for accurate diagnosis [11]. Abnormal cytology was observed in 18.7% of participants, including ASCUS, LSIL, HSIL and squamous cell carcinoma, which is slightly lower than rates reported in other studies [8]. Variations may result from differences in population characteristics and screening protocols [12].

Colposcopic examination revealed abnormal findings in 42.7% of cases, comparable to previous findings where abnormal colposcopic impressions were noted in approximately 43% of women with unhealthy cervix [5]. This highlights colposcopy's ability to detect epithelial changes not always identified by cytology [13]. Histopathology confirmed precancerous or cancerous lesions in 53.1% of women with abnormal colposcopic findings, similar to studies reporting about 50% detection rates of epithelial abnormalities on biopsy in such populations [5]. The correlation between colposcopic impressions and histopathological diagnoses showed substantial agreement (κ = 0.78), with 90.9% concordance, consistent with prior research demonstrating high correlation rates between these diagnostic modalities [8, 14]. Colposcopy demonstrated higher sensitivity (83.3%) but lower specificity (72.7%) compared to Pap smear (sensitivity 54.3%, specificity 96.2%), echoing findings from previous comparative analyses [5, 6 and 15]. This indicates colposcopy's strength in minimizing false negatives, though at the cost of increased false positives, potentially leading to unnecessary interventions [7]. Despite a slightly lower positive predictive value (88.9%) than Pap smear (91.7%), colposcopy showed higher overall diagnostic accuracy (82.7% vs. 79.3%), reinforcing its value as a reliable diagnostic tool when integrated with cytology for comprehensive cervical assessment [8, 15]. Cytology can be an acknowledged procedure of screening for cervical neoplasia, and that the worth of colposcopy was known mainly from the evaluation of patients with abnormal cervical smears [16].

## Conclusion:

Colposcopy offers higher sensitivity than Pap smear for detecting cervical abnormalities in women with clinically unhealthy cervix. Its strong correlation with histopathology supports its diagnostic reliability. A combined Pap smear and colposcopy approach enhances detection, especially in resource-limited settings.
